# The barriers and facilitators to the implementation of clinical guidance in elective orthopaedic surgery: a qualitative study protocol

**DOI:** 10.1186/s13012-015-0273-6

**Published:** 2015-06-02

**Authors:** Amy Grove, Aileen Clarke, Graeme Currie

**Affiliations:** Division of Health Sciences, Warwick Medical School, University of Warwick, Coventry, UK; Entrepreneurship & Innovation, Organising Healthcare Research Network, Warwick Business School, University of Warwick, Coventry, UK

**Keywords:** Guidelines, Orthopaedic surgery, Evidence-based medicine, Comparative case study, Barriers, Facilitators, Implementation

## Abstract

**Background:**

Clinical guidelines in orthopaedic surgery aim to improve the efficiency, quality and outcomes of patient care by ensuring that treatment recommendations are based on the best available evidence. The simple provision of guidelines, however, does not ensure fidelity or guarantee their uptake and use in surgical practice. Research exploring the factors that affect surgeons’ use of evidence and guidelines has focused on understanding what evidence exists for current clinical decisions. This narrowed scope emphasises the technical, educational and accessibility issues but overlooks wider factors that help explain how and why guidelines are not implemented and used in surgery. It is also important to understand how we can encourage the implementation processes in practice.

By taking a social science perspective to examine orthopaedic surgery, we move beyond the narrow focus and explore how and why clinical guidelines struggle to achieve full uptake. We aim to explore guideline uptake to discover the factors that contribute to, or complicate, appropriate implementation in this field. We need to go beyond traditional views and experimental methods to examine the barriers and facilitators of implementation in real-life NHS surgical practice. These could be multifactorial, linked to individual, organisational or contextual influences, which act on the guideline implementation process.

**Methods/design:**

We will use ethnographic methods to conduct case studies in three English NHS hospitals. Within each case, we will conduct observations, interviews and analysis of key documents to understand experiences, complex processes and decisions made and the role of clinical guidance and other sources of evidence within orthopaedic surgery. The data will be transcribed and analysed thematically. Comparisons will be made within cases and across cases.

**Discussion:**

Guidelines are a fundamental part of clinical practice, and various factors must be considered when preparing for their successful implementation into organisations. Understanding the views and experiences of a range of surgical, clerical and managerial staff across multiple orthopaedic departments will capture the complexity and variety of factors that can influence surgical decisions. The findings of our study will identify the specific features of orthopaedic practice to help guide the development of strategies to facilitate guideline uptake in everyday surgical work.

## Background

Evidence-based medicine (EBM) is defined as the integration of clinical expertise with evidence from research [1] to improve the efficiency, quality and outcomes of patient care by ensuring that treatment recommendations are based on the best available clinical evidence. The attempted mobilisation of research evidence into practice in the UK has traditionally occurred through the dissemination of clinical guidelines into the National Health Service (NHS) such as those produced by the National Institute for Health and Care Excellence (NICE) [2] and the Scottish Intercollegiate Guidelines Network (SIGN) [3]. A key remit for these policy makers is to ensure the full implementation of clinical guidelines [4]. The actual level of implementation has been, and can be, further examined using traditional experimental methods, for example, to provide a rate of uptake or impact through educational strategies or audit [5]. Several systematic reviews on universal guideline implementation have been conducted but provide limited insight on how to choose implementation strategies that address implementation problems [6–9]. This type of research typically demonstrates limited success of guideline implementation and evidence-based knowledge into clinical practice, particularly in community care services [6, 10–14]. To move beyond this and explore how and why clinical guidelines struggle to achieve full uptake and use within surgery, we need to go beyond traditional views and experimental methods to examine the barriers and facilitators of implementation in real-life NHS practice. These barriers and facilitators could be multifactorial, linked to individual, organisational or contextual influences, which act on the guideline implementation process [6–9] but could also directly stem from the professional work of surgeons.

The determinants of successful implementation reflect not only the people involved but their roles, their positions in the organisation and the epistemic communities to which they belong [14, 15]. Producers and users of evidence often sit within different epistemic communities with varying knowledge domains. Therefore, it can be difficult to integrate knowledge and evidence across the social, scientific and clinical communities [15]. This may be the case in clinical guideline implementation, as what determines evidence itself is often contested by those involved in its production and use [14–17]. The different sources of evidence, for example, guidelines or personal experience, are ranked differently and interpreted as more or less important in practice. In the day-to-day work of clinical professionals, such as orthopaedic surgeons, there are many forms of evidence and knowledge which play an important part in decision-making processes.

This is complicated by the local, contextual and social circumstances within which clinical decisions are made as the processes within organisations and the resources available are all important [18].

Maintaining clinical autonomy and medical judgement are also seen as important, and guidelines which conflict with traditional practice or run against the norms of the clinical group or organisation may encounter resistance [16, 19]. More powerful sources of influence stem from the evidence and knowledge derived from clinical communities of practice within which surgeons work [20, 21]. These are guided by internalised, collective and tacit guidelines built up over time across networks of colleagues and important clinical experts [10]. These additional sources can facilitate or hinder implementation of guidelines such as those produced by NICE [1]. This raises the question of how surgical work practices change, perhaps partially or not at all, when orthopaedic surgeons are faced with new and potentially competing evidence sources. The aim of our research is to explore these implementation problems through the application of ethnographic methods to investigate the barriers and facilitators to the use of evidence-based clinical guidelines in decisions made in elective orthopaedic surgery in the NHS.

### Exemplary case: orthopaedic surgery

Orthopaedic surgery as a speciality represents a highly professionalised area of clinical work where an elite community of practice is strongly embedded [12]. The depth of insight gained from the study of these highly professionalised clinical groups will offer a distinctive perspective on the challenges of getting research into surgical practice.

This group of professionals has been shown to retain substantial autonomy over their work practices and tend to resist external intervention [22–24]. The implementation of clinical guidance produced outside this community is likely to be complex and fraught with challenges. Surgery is often seen as a craft developed through years of apprenticeship-style training and experience, rather than as a science which can be codified and written into guidelines [12]. The issue of craft versus science in orthopaedic surgery may constitute one of a range of factors which may facilitate or hinder implementation. Investigating these unexplored questions can offer greater insights compared to the simple reporting of guideline uptake, although these complex problems require distinctive methods to examine the key issues.

In order to bring more clarity to this area, and to inform our research as a whole through conceptual framework development, we will conduct a systematic review of published evidence to identify the different sources of evidence and knowledge which have been reported as important for decision-making specifically within orthopaedic surgery. Whereas previous reviews have provided general or condition-specific summaries of evidence [6–9], we will build on previous studies by considering the views of multiple health professionals within the orthopaedic community as well as clerical staff and managers and will examine orthopaedic departments in hospitals with different organisational structures.

## Methods/design

To explore the ‘how’ and ‘why’ questions of implementation within orthopaedic surgery, we will take a social science rather than clinical science perspective [25–27]. We will conduct comparative qualitative case studies undertaking comparisons across multiple cases and at multiple levels within each case [28]. We will examine our data for examples of surgeons’ values, beliefs and norms about forms of evidence, such as clinical guidelines, and how these are used in practice. We will take a wider perspective to investigate how organisations support clinical guideline implementation in surgery and the contextual factors impeding or facilitating the use of this particular type of knowledge.

Our case study design involves three NHS hospital departments in England selected to show variation in their proximity to and association to an academic institution and by implication to an academic research environment. Drawing on our earlier reviews of the literature, we observed a strong relationship between academic and non-academic clinicians and their use of evidence in practice. To investigate this further, we selected one orthopaedic department in a teaching hospital, one in a non-teaching hospital and another designated academic orthopaedic department where the members hold hybrid academic/clinical roles in both the trust and the local university.

We will follow the roadmap for case study research developed by Eisenhardt [29]. Research will be started as close as possible to the ideal of *no theory under consideration* since pre-selected theoretical perspectives may limit the transferability of our research findings [29]. Nevertheless, our approach to data collection and analysis will combine both induction and deduction as we will be informed by the review of literature and scoping work [30]. We do not set out to answer a specific hypothesis. Data collection in the field will allow concepts of interest to develop as the work progresses [31, 32].

Each case will comprise of ethnographic data collection methods including document analysis, observations and interviews. The research aligns to a constructivist understanding since we will seek to explore the meanings that groups of surgical staff and affiliated health professionals hold towards different forms and sources of evidence and the influence of clinical guidelines. We will follow progressive focusing, in that what we learn as the data collection progresses will guide and inform the next stages of data collection [33, 34]. For example, we may choose to investigate the role of clinical audit teams in guideline implementation if they are consistently mentioned during interviews or present during observations. This flexible approach is a key feature of the case study design which will allow us to adjust the data collection processes to further investigate emergent themes and to take advantage of opportunities which may arise [29].

### Setting and participants

We will use purposive sampling to select the first participants at each hospital site and then use snowball sampling to select further individuals to interview as the study advances. The aim is to achieve a maximal variation sample of orthopaedic professionals who conduct or facilitate joint replacement surgery, i.e. we are sampling for heterogeneity of professional background, level of training and years in practice [35]. We will approach administrators and managers who are involved in decisions made for patients undergoing hip replacement surgery. The aim of this is to see how the phenomenon under investigation is seen and understood among different professional groups in different workplace settings and across three differing hospital departments. These individuals will help to expand upon and enhance our understanding and further develop our findings [32]. We will continue to observe and interview participants until no new information is obtained from the data, and theoretical saturation has been reached [36]. We will compare data collected from these groups across the three cases to identify differences and similarities in the way evidence is used and any barriers and facilitators to implementation that have become apparent.

### Case study sites

Case study site A is specifically designated as an academic orthopaedic department and orthopaedic trauma centre linked to an Academic Health Science Network. The department works in partnership with the local university medical school and is also a teaching hospital. There is an integrated clinical academic training scheme in orthopaedics. The research team conducts effectiveness and cost-effectiveness studies, mainly national randomised control trials (RCTs), of various techniques and treatments within orthopaedic surgery. The clinical service provides specialist musculoskeletal care alongside the departments of rheumatology, physiotherapy, radiology and paediatrics. The clinical staff comprise consultants, clinical fellows, specialist registrars and allied health professionals (AHPs). Many of the surgeons hold joint academic/clinical posts at the trust and the university. In 2014, the trust performed approximately 580 hip procedures [37]; of these, 93–97 % used Orthopaedic Device Evaluation Panel (ODEP)-rated implants [38]. ODEP is an organisation established in 2002 to monitor NICE guidance on hip implants. They assess implants against benchmarks set by NICE for implant survivorship.

Case study site B is a large orthopaedic department in a teaching hospital trust with a specialist trauma centre. Unlike site A, it is not a designated academic orthopaedic department with a specific clinical academic training scheme. It is one of the largest orthopaedic surgery units in England and receives national referrals for complex hip implant revision surgery. The clinical department provides a range of orthopaedic treatments and surgery delivered by a multidisciplinary team of specialists. Site B is also a teaching hospital and has a small academic team that carries out research, development and training; for example, site B acts as a ‘spoke’ data collection site in national RCTs. In 2014, site B performed approximately 573 hip procedures [37]; of these, 92–97 % used ODEP-rated implants [38]. Clinical professionals at site B may or may not participate in academic work depending on their own capacity and willingness.

Case study site C is a small district general hospital trust with a specialist practitioner interface service. This site is not affiliated to an orthopaedic academic department or university and is not a teaching hospital. The clinical team provides general orthopaedic services to the local population and is supported by a group of designated allied health professionals who provide a specialist musculoskeletal assessment interface between general practitioners, patients and the orthopaedic department. Site C performed approximately 716 hip procedures in 2013 [37], and 55–100 % of these procedures used ODEP-rated implants [38].

We will observe and interview orthopaedic surgeons, AHPs, orthopaedic nurses, clerical staff and members of the hospital governance boards and relevant committees. The aim of the interviews is to understand perspectives and experiences of a diverse sample of professionals working in and across orthopaedic health services. Within each case, observations will be used for two reasons: firstly to understand the complex processes and decisions made using clinical guidance or other sources of evidence and knowledge within each orthopaedic department and secondly to allow us to triangulate interview data from different participants and with observational data [39].

The planned interview sample is detailed in Table 1. As mentioned previously, purposive sampling will be used to obtain the initial sample and then snowball sampling will be used throughout the remaining data collection process. This will be based on the results of the analyses and the progressive focussing we wish to perform. It may be that certain individuals will be re-interviewed if their role and experience necessitate further reflection or explanation. To complement and elaborate on the case study data, we will conduct key stakeholder interviews with national experts in the field and individuals who represent policy-making organisations such as local clinical commissioning groups, NICE, the British Orthopaedic Association and British Hip Society.

**Table 1:** Planned sample in each case study

Setting	Clerical staff		Managers		Clinical professionals	
	Organisational level	Department level	Organisational level	Department level	Nurses/AHPs	Surgeons
A	2	2	2	2	5	7
B	2	2	2	2	5	7
C	2	2	2	2	5	7
Subtotal	6	6	6	6	15	21
Total	60					

### Data collection

Data collection at each site will consist of a combination of ethnographic methods including the following: document analysis, observation and interviews. Observations will consist of opportunistic shadowing on the wards, watching clinic and teaching sessions and attendance at planned operating sessions, particularly the pre-theatre preparation time. Other parts of daily work will be observed including meetings and seminars, lunch and break periods and *ad hoc* teaching of junior staff. During this time, we will seek out examples which demonstrate how the use of clinical guidelines are hindered or facilitated within the department. Observations also allow for more informal discussion with the surgeons and clinical staff as they go about their daily tasks and provide the opportunity to ask why certain actions or processes occur.

The interviews will examine the views of the individual and their perceptions about their clinical community and hospital departments relating to the use of clinical guidelines. Suggested topics are displayed in Table 2. Interviews will not follow a predefined structure as this may limit the research findings; we will continuously develop interview topics as the data collection progresses.

**Table 2:** Planned topics for discussion during interview

Interview topics	Descriptions of the general approach to practice and how clinicians approach treatment decisions
	Discussion about the sources of evidence and knowledge that influence practice in general
	Participants’ beliefs and experiences of using or having contact with clinical guidance (NICE in particular),
	Participants’ views regarding how EBM and clinical guidelines could be better mobilised into practice

Each potential participant will be contacted by the lead researcher (AG) and asked to participate in the study. The interviews will be conducted by the same researcher in hospital or university buildings. It is anticipated that each interview will take between 30 and 60 min. They will be tape recorded and transcribed verbatim by the lead researcher (AG) and uploaded into NVivo software (version 10) [40] to assist in the data manipulation during analysis.

We anticipate the biggest challenge in the study is access in general and more specifically the lead researcher being ‘in the right place at the right time’ to observe key events and speak to key people. It will be important to schedule the interviews in a manner that is acceptable to the busy clinical professional which does not disrupt normal practice but allows it to be observed. The interviewer will ensure that the interviews are as informal and discussion-like as possible so that the participants can feel free to discuss their beliefs and experience of evidence and guideline use in practice without fear of judgement or reprimand. The participants will be informed that the lead researcher and the wider team are impartial and have set out to understand what normal practice is for staff working in or affiliated to orthopaedic surgery in the NHS.

Additional relevant documentation will be collected for document analysis. Documents may include government policy documents, national and local clinical guidance and published information about the cases such as annual reports and quality monitoring reports. Document analysis will help us to understand and frame the intentions of practice change within the orthopaedic departments and provide a wider understanding of the context within which decisions are made. We will attempt to understand each case individually and in as much depth as is feasible. We will take advantage of the flexible data collection methods and make adjustments where required during the process to allow for progressive focusing. During this time, the data collection will remain systematic and transparent; decisions will be recorded in case summaries and field notes.

### Data analysis

The three sources of data from each case study will be processed into text format databases before analysis begins. We will analyse the texts and interview transcripts using thematic analysis taking an inductive (data-driven) and deductive (guided by the review of literature) approach. The process of thematic analysis follows various stages which occur sequentially, but the process is often iterative [41]. Stages include transcription data familiarisation, coding and development of categories from codes. The coding process will be conducted by the research team (AC, AC, GC); the categories will be interpreted and developed into the key themes of research. This final step involves the recurrent identification and comparison of themes both within and across subcategories and development of broader categories to develop a narrative which describes the barriers and facilitators to the implementation of clinical guidelines in orthopaedic practice. Themes will be compared and contrasted with the conceptual framework developed from the systematic literature review.

### Case studies

Each case will be written up in detail to develop real-life depictions of clinical guideline evidence use in orthopaedic surgery decision-making. These individually will be pure descriptions of the current situation but are central to generating insight [30]. The aim of this step is to become familiar with each case as a stand-alone entity. This process will allow us to see patterns in each case as they emerge but also will provide familiarity with each case to accelerate the cross-case comparisons [29]. The aim of the cross-case comparison is to search for further patterns in the data; for example, we will select categories such as professional role types and look for within-group similarities and with-intergroup differences and compare these with other cases and also with existing literature. It will be important at this stage not to develop premature and false conclusions by examining the data in different ways [42], for example, taking the individual- and departmental-level perspective and comparing it to findings from a wider organisational perspective.

Following this, we will compare data by data collection method to investigate the unique insights obtained from the different collection methods. This will enable us to investigate whether findings from the interviews are supported by observation or document analysis and *vice versa*. Triangulation across data collection methods will help to facilitate the validation of data through cross verification from more than two sources [39]. The data collected will not contain identifiable characteristics. Each participant will be coded, for example, participant a1, b2, c3. The study data will be retained for the life of the research project and 1 year following completion. The data will be stored in electronic format in password-protected files.

### Ethics and funding

The study was approved by the Biomedical and Scientific Research Ethics Committee (BSREC) of The University of Warwick, England (approved June 2nd 2014; reference number REGO-2014-645) and the research and development departments of each of the three hospital sites (site A approved 30.06.2014, site B approved 23.10.2014, site C approved 21.08.2014). In line with the ethical approval, each participant will be asked to sign an informed consent form as will be provided with information outlining the purpose of the study and their rights to withdraw. Confidentiality is protected as the data will be anonymised.

This work is supported by the National Institute for Health Research, Doctoral Fellowship Programme (2013-06-064). Funding sources had no role in the research once the grant had been awarded.

## Discussion

Clinical guidelines in orthopaedic surgery are an important part of clinical practice to help drive improvement, guarantee safety and ensure that NHS resources are used responsibly [43]. Empirical research has demonstrated that clinical scientists, such as orthopaedic surgeons, privilege the experimental method and randomised control trials (RCTs) and therefore value this type of research over more theoretical social sciences [15]. Nevertheless, the implementation of clinical guidelines even when based on the findings of empirical research including gold standard RCTs is not guaranteed in surgical practice. This suggests that well-developed guidelines are necessary but not sufficient to achieve the goals of organisations such as NICE and SIGN when applied to the surgical specialties.

By taking a social science perspective to examine this traditionally highly specialised area of orthopaedics, we seek to discover and explain this gap in the process of knowledge mobilisation for clinical guidelines in surgery. Unlike RCTs and experiments, this perspective will allow us to uncover and explain the barriers and facilitators to guideline implementation and use in orthopaedics. We anticipate that the different types of knowledge that exist in practice, for example, clinical evidence, experiential knowledge of the ‘art’ of surgery, apprenticeship-model training and the importance that surgeons and departments attach to them, will interact with the varying contextual factors to produce variation in the extent to which guidelines are implemented and used. We set out to discover how this multitude of factors will support or hinder the implementation process.

### Strengths and limitations

The key strength of our research is the use of multiple sources of data (interviews, observations and document analysis) to study the same phenomenon. The combination of these multiple qualitative methods will enable us to overcome the weakness that comes from a single method [44]. The triangulation of data collected using these three methods will enhance the credibility of our analysis and findings; when the evidence from one source corroborates a pattern from another, the finding is stronger and better grounded [29]. If the data from different methods conflict, or appear as negative cases, this will provide us with an interesting opportunity to investigate the meaning behind the differences. This comparative process will lead to a more sophisticated understanding of our data [28, 29].

Our study has limitations, mainly that the data will be collected in each case through documents, interviews and observation of clinical and non-clinical staff performing their day-to-day activities. The direct observation of health professionals in their practice may drive ‘good’ behaviour, known as the observer effect [45]. To ensure the quality and rigour of our data, we are extending our access and observation as much as possible, whilst also conducting crosschecks and validation during interviews and between different individuals.

## Conclusion

The aim of our research is to explore implementation problems through the application of ethnographic methods to investigate the barriers and facilitators to the use of evidence-based clinical guidelines in decisions made in elective orthopaedic surgery in the NHS. The results of our study will highlight the range of complex and competing sources of evidence and knowledge which influence the work practices of surgeons in the NHS. Through this research, we will provide a clear but rich picture of the barriers and facilitators to the use of evidence-based clinical guidelines specifically for orthopaedic surgery in the NHS. The inclusion of a diverse range of professionals and frontline surgeons and clinicians will capture the complexity of the roles and responsibilities that influence guideline uptake. The results of this study will, we hope, guide the development of interventions that are grounded in the data and aimed at improving the introduction and implementation of guidelines into surgical practice. It is important to identity barriers and choose to tailor implementation strategies to fit so that the outcomes can be optimised [6]. Future studies will focus on design and testing of these interventions to ensure the implementation strategies match the identified barriers and facilitators. This will mean that the solutions can work ‘in the hands’ of surgeons, doctors and service managers.

## Abbreviations

AHPs: allied health professionals
BSREC: Biomedical and Scientific Research Ethics Committee
EBM: evidence-based medicine
NHS: National Health Service
ODEP: Orthopaedic Device Evaluation Panel
RCTs: randomised control trials
SIGN: Scottish Intercollegiate Guidelines Network
